# Natural Ageing of PLA Filaments, Can It Be Frozen?

**DOI:** 10.3390/polym14163361

**Published:** 2022-08-17

**Authors:** Jaime Orellana-Barrasa, Ana Ferrández-Montero, Begoña Ferrari, José Ygnacio Pastor

**Affiliations:** 1 Centro de Investigación en Materiales Estructurales (CIME), Universidad Politécnica de Madrid, 28040 Madrid, Spain; 2 Instituto de Cerámica y Vidrio (CSIC), Campus de Cantoblanco, 28049 Madrid, Spain

**Keywords:** PLA, fused filament fabrication, thermal and mechanical properties, ageing, freezing, desiccant

## Abstract

The physical ageing of polylactic acid (PLA) is a phenomenon that changes the material’s properties over time. This ageing process is highly dependent on ambient variables, such as temperature and humidity. For PLA, the ageing is noticeable even at room temperatures, a process commonly referred to as natural ageing. Stopping the ageing by freezing the material can be helpful to preserve the properties of the PLA and stabilise it at any time during its storage until it is required for testing. However, it is essential to demonstrate that the PLA’s mechanical properties are not degraded after defrosting the samples. Four different methods for stopping the ageing (anti-ageing processes) are analysed in this paper—all based on freezing and defrosting the PLA samples. We determine the temperature and ambient water vapor influence during the freezing and defrosting process using desiccant and zip bags. The material form selected is PLA filaments (no bulk material or scaffold structures) printed at 190 °C with diameters between 400 and 550 µm and frozen at −24 °C in the presence or absence of a desiccant. The impact of the anti-ageing processes on PLA’s ageing and mechanical integrity is studied regarding the thermal, mechanical and fractographical properties. In conclusion, an anti-ageing process is defined to successfully stop the natural ageing of the PLA for an indefinite length of time. This process does not affect the mechanical properties or the structural integrity of the PLA. As a result, large quantities of this material can be produced in a single batch and be safely stored to be later characterised under the same manufacturing and ageing conditions, which is currently a limiting factor from an experimental point of view as polymeric filament properties can show significant variety from batch to batch.

## 1. Introduction

PLA is the most common polymeric material found in the additive manufacturing industry, and it is printed via the material thermal extrusion process or material extrusion [1,2,3,4,5]. This material extrusion process consists of three main parts: melting, extrusion and cooling. The melting process liquefies the PLA and consequently removes any previous thermal history (however, thermal degradation might occur at this stage) [6]; the extrusion process generates filaments with the desired shape as the material flows through the nozzle; and finally, the cooling process solidifies the share of the extruded filament.

This last part generates an out-of-equilibrium microstructure inside the PLA, as the polymer chains of the amorphous phase are frozen in non-equilibrium positions with high enthalpy values compared with its stable state [7,8,9,10]. This excess of enthalpy is lost through ageing as the polymeric chains find lower energy configurations due to the thermal motions and can be assessed with increased enthalpic relaxation in differential scanning calorimetry.

Several ageing mechanisms are described in the bibliography, such as hydrolysis [11,12,13,14,15] or photodegradation [16,17,18,19] among other mechanisms [20]. However, the one related to the evolution of the non-equilibrium state after cooling is the physical or natural ageing, a type of ageing that occurs due to the macromolecular movements of the polymeric chains into more stable configurations employing the thermal energy available in the material at temperatures below the glass transition (T_g_) [21,22,23]. Slowly after the cooling process, these polymeric chains rearrange into more stable configurations, which changes the material’s properties, such as the glass transition (T_g_), the yield strength or the enthalpic relaxation [24]. This ageing process happens at temperatures below T_g_ but faster when the temperature is closer to T_g_ [25,26].

As is well known and from our previous experience [24], T_g_ for the PLA is between 50 to 63 °C, which are temperatures relatively close to room temperature, making it even more important to understand the ageing of the PLA and how to stop it when needed. The rearrangement of these polymer chains at the molecular level is the mechanism for physical ageing [27], and it is desired to stop these polymeric chain movements. Thus, the PLA was frozen, decreasing the thermal energy available in the system.

One of the PLA ageing mechanisms is hydrolysis, making the PLA’s ageing highly susceptible to ambient humidity. For this reason, we studied the influence of adding a desiccant in different steps during the anti-ageing process. Although the temperatures in this work are below 50 °C and were performed in dry weather (which allows us to neglect the influence of the humidity moisture on the ageing due to hydrolysis [28]), we want to analyse the effect of humidity on the mechanical properties of the PLA during freezing to stop the ageing process.

Our research aims to study the mechanical behavior of the PLA as it is intended to be used for medical prostheses, and it will be helpful to store the PLA and stop the ageing so that we can adapt the research schedule to external factors, such as COVID-19, without having to restart all the experiments. The value and evolution of the enthalpic relaxation usually indicate how aged the polymer is, which can be easily quantified by differential scanning calorimetry (DSC) [28,29].

The DSC provides plenty of information regarding the material’s thermal properties. However, at the same time, it also includes information on the physical properties, such as the residual monomer after a polymerisation curing reaction [30], the crystallisation or the ageing of the material. For our research, it is critical to demonstrate that the mechanical properties are not affected by these freezing–defrosting anti-ageing processes.

## 2. Materials and Methods

**Figure 1:**
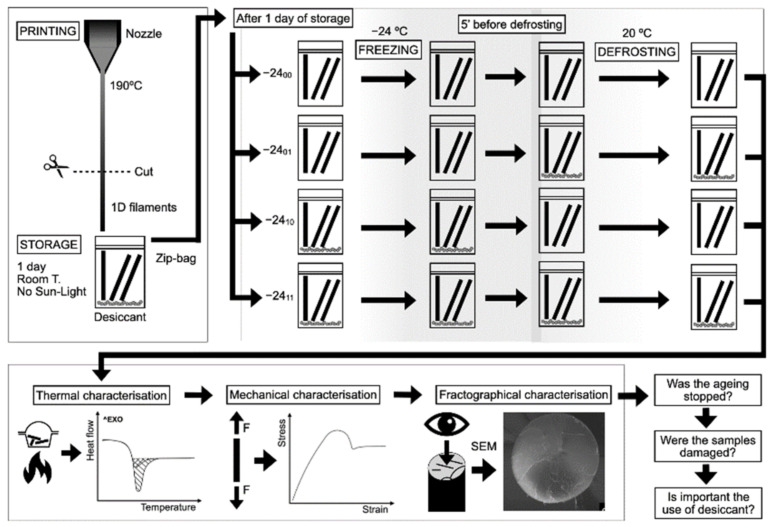
Summary of the materials and methods: material preparation, anti-ageing process, thermal, mechanical and fractographical analysis.

A summary of the materials and methods is presented in Figure 1.

### 2.1. Material Preparation

The PLA studied was the Ingeo biopolymer 2003D (Nature Works, United States), a high-molecular-weight polymer derived from natural sources and composed of 96% l-lactide and 4% d-lactide. This PLA was printed (extruded) as single filaments or 1D structures, as schematised in Figure 1, at the Institute of Glass and Ceramic (ICV, CSIC, Madrid, Spain) in a 3D printer Fused Filament Fabrication (Original Prusa i3 MK3S+ from Prusa Research, Prague, Czech Republic) at 190 ± 1 °C with a 0.4 mm nozzle, following the methods described in [24]. Filaments with a diameter between 400 and 550 µm were obtained.

The degradation by hydrolysis of the PLA molecular weight (M_w_) was prevented by maintaining the material in low humidity and low-temperature conditions [31]. The low humidity condition was obtained by storing the samples inside PET zip-bags with silica desiccant inside. We observed how the color indicator of the silica desiccant inside the zip-bag remained stable over several weeks instead of only minutes. All the printed samples were dimensionally characterised with a Nikon Profile Projector V-12B, within a resolution of ±1 µm. The filaments with the right consistency on the diameter were studied, as this is essential for mechanical characterisation.

### 2.2. Anti-Ageing Methods

Printed PLA filaments were stored inside PET zip bags. Four different anti-ageing methods based on four freezing–defrosting routes were studied, as schematised in Figure 1 and described in more detail in Table 1. The influence of the presence of desiccant inside the zip bags during the freezing and defrosting processes was studied. A reference material aged at room temperature inside zip bags with desiccant was also studied.

The research was conducted in Madrid with dry weather and a maximum humidity percentage at room conditions below 70%. Samples were aged from 1 day to 273 days (9 months). Regarding [28], PLA degradation driven by hydrolysis requires temperatures of 50 °C, and thus it is not expected to occur on the PLA aged at room temperature.

The effectiveness of the anti-ageing method was assessed with the results from the Differential Scanning Calorimetry (DSC). Furthermore, tensile tests were performed to ensure that the mechanical properties of the filaments have not decreased nor increased and thus remained stable. Obtaining stable properties, especially for enthalpic relaxation and mechanical properties, indicated a successful anti-ageing method.

### 2.3. Thermal Analysis

Thermal analysis was conducted using the DSC technique on a Mettler Toledo 822^e^. The temperature range of this analysis was from 40 to 180 °C, at a heating rate of 10 °C/min. The weights of the samples were between 5 to 10 mg, and the filaments were cut into pieces of 3–4 mm, which were placed inside the DSC crucible. Before the tests, the DSC apparatus was calibrated with the Indium standard. A direct comparison between the DSC graphs was performed to identify any variation between the samples aged at room temperature and the frozen samples.

The evolution of the glass transition, the enthalpic relaxation temperature, and the enthalpic relaxation enthalpy were fitted to the Kohlraushch–William–Watts (KWW) [32]. This followed the same procedure as the previous study [24].

### 2.4. Mechanical Characterisation and Fractography

**Figure 2:**
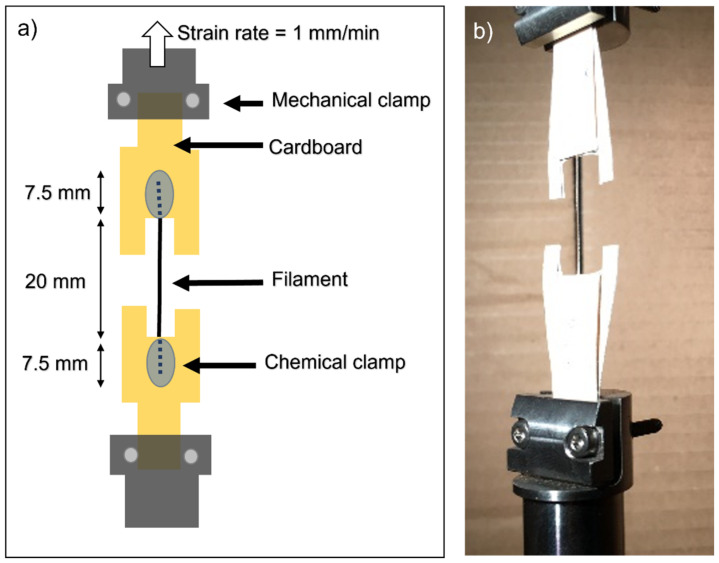
(**a**) Scheme of the tensile test sample and clamping methods. (**b**) Picture of the actual tensile test sample.

Samples were defrosted for one hour at room temperature before performing the mechanical testing. Tensile tests were prepared according to the ISO 527-3:2019 and served in the universal testing machine INSTRON 5866 (Instron, Norwood, MA, United States). A 1 kN cell was used for measuring the forces. Samples with a 20 mm length were tested at 1 mm/min (strain rate of 5 × 10^−2^ min^−1^) at ambient humidity and room temperature. To avoid damage to the filaments due to the mechanical clamps, filaments were chemically clamped to cardboard with a cyanoacrylate glue following the procedure described in [24] and schematised in Figure 2. The yield strength, elongation at break and the elastic modulus were calculated. The formation or absence of a necking was studied as well. Fractographies of samples were taken with an AURIGA FESEM ZEISS (Carl Zeiss Microscopy GmbH, Germany) with a 20 nm thickness carbon coating in a LEICA EM ACE200 Vacuum Coater (Leica Myscrosystems, Germany) before the FESEM following the methods in [24].

The yield strength and the elastic modulus were fitted to a logistic model, as developed in [24].

## 3. Results and Discussion

### 3.1. Morphology

**Figure 3:**
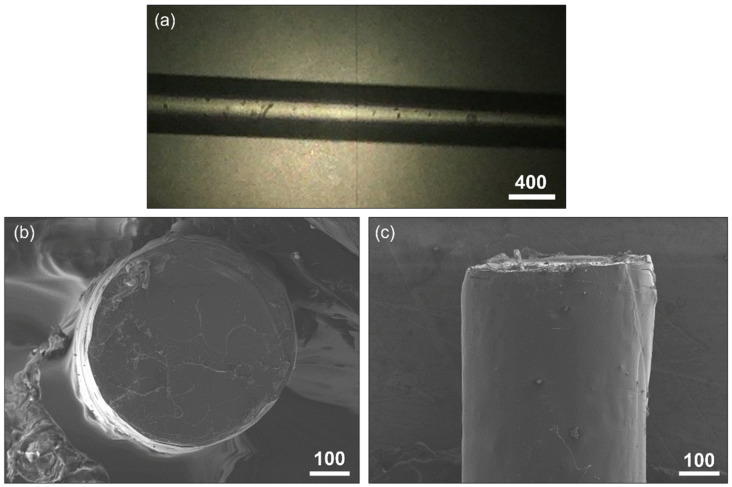
(**a**) Detail of the PLA filament with the profilometer. (**b**) Fractography of the filament with the SEM in which the excellent ovality can be observed. (**c**) Transversal view of the same broken filament. Scale bars in microns.

Samples extruded had a diameter between 400 and 550 µm, varying smoothly along the length of the filaments with high stability on the diameter through the filaments and good ovality, as shown in Figure 3.

### 3.2. Thermal Properties

All the results from the thermal properties are shown in Table 2.

**Figure 4:**
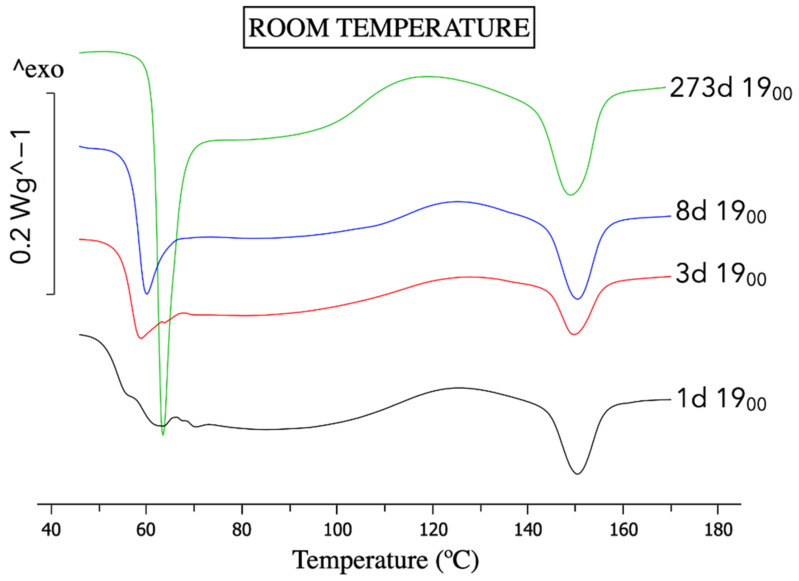
DSC graphs for samples aged at room temperature using the nomenclature in Table 1.

The ageing at room temperature on the reference material has produced an intense increase on enthalpic relaxation, indicative of physical ageing and a shift of T_g_, as shown in Figure 4, which is coherent with our previous study [24]. This proves that the material studied has the potential to age.

**Figure 5:**
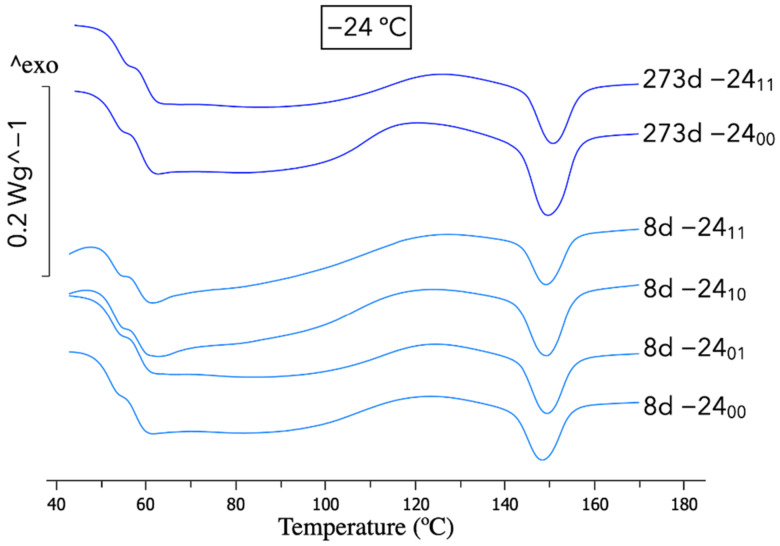
DSC graphs for samples aged at −24 °C following the nomenclature in Table 1.

However, no changes were observed on samples frozen at −24 °C, regarding the results of the DSC in Figure 5, indicating that −24 °C stopped the physical ageing. Our previous work provides a deeper analysis of the DSC changes with physical ageing [24].

As can also be observed in the DSC diagram in Figure 5, for samples frozen at −24 °C there is no influence of using desiccant during the anti-ageing process. Neglectable differences can be found by comparing all the curves of the frozen samples.

With these results, it can be ensured that freezing the PLA at −24 °C has effectively stopped the PLA ageing. The fact that the ageing was arrested by decreasing the temperature is coherent with the ageing mechanism that requires thermal energy for the motion of the polymeric chains into those more stable configurations that produce a loss of enthalpy.

**Figure 6:**
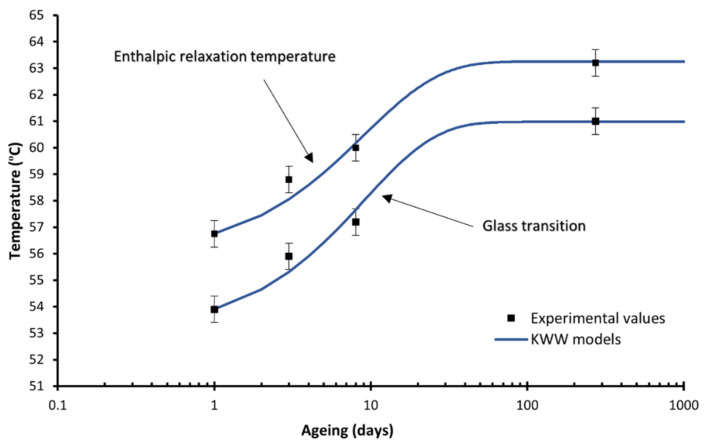
Glass transition and enthalpic relaxation temperatures for the material 19_00_ with the corresponding KWW fitting models (lines). The line represents the KWW model, the dots the experimental data.

**Figure 7:**
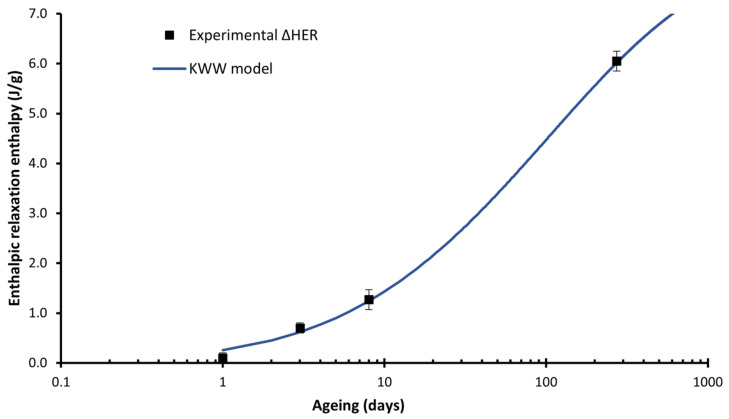
Enthalpic relaxation enthalpy for the material 19_00_ and the corresponding KWW fitting model (line). The line represents the KWW model, the dots the experimental data.

A deeper analysis of the DSC results in Figure 6 and Figure 7 represents the modelling of the glass transition, the enthalpic relaxation temperature, and the enthalpic relaxation enthalpy with the KWW model with a remarkable agreement with the experimental data. The KWW parameters obtained for the different models are as follows:

For the glass transition and the enthalpic relaxation temperature, the values are β = 8.00 and $\tau_{0}$ = 0.9 days, and β = 7.00 and $\tau_{0}$ = 0.82 days respectively, which plots are represented in Figure 6. It is observed how the stabilisation time for these two properties for the filaments aged at room temperature is of two months, coherent with our previous results [24].

For the enthalpic relaxation enthalpy: β = 0.27 and $\tau_{0}$ = 0.24 days, which plot is represented in Figure 7, indicates that the enthalpic relaxation enthalpy will continue growing after 273 days on the material aged at room temperature, which is coherent with our previous study [24].

It is fascinating that the ratio between the β and $\tau_{0}$ parameters on the KWW model is around 10 for both the glass temperature and the enthalpic relaxation temperature, which are similar to the ones obtained in our previous study, indicating that this relation for these properties could be a constant for the material PLA 2003D.

Regarding the crystallinity values, neglectable crystallinity percentages were calculated from the DSC, again coherent with our previous study of this material [24].

### 3.3. Mechanical Behaviour

Once it was obtained that the ageing can be stopped by freezing the PLA at −24 °C, it is crucial to see if the freezing–defrosting process has damaged the material. The mechanical properties were studied by an uniaxial tensile test, as they are susceptible to any induced microstructural damage.

#### 3.3.1. Yield Strength

**Figure 8:**
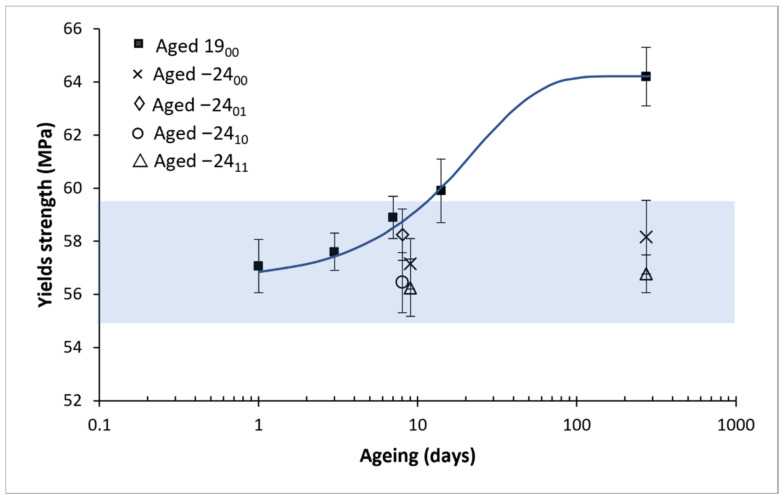
The average value of the yield strength with its mean square error. The reference material aged at room temperature is with bold marks and with its logistic fitting (line); empty symbols are from frozen and defrosted materials. For better visualisation, samples aged 9 days are separated in 8 and 9 days in the graph, but all do correspond with 9 days. Six samples were measured for each ageing conditions and error bars are the standard deviation.

**Figure 9:**
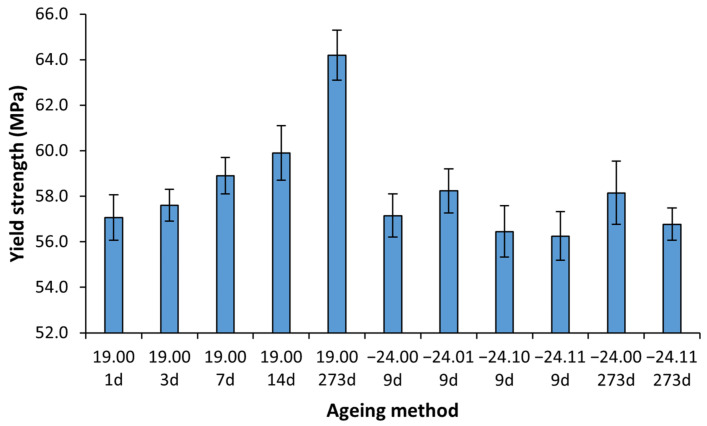
Bar diagram with the average value of the yield strength with its mean square error versus the ageing time. The Shadow area shows the frozen sample value interval. Six samples were measured for each ageing conditions and error bars are the standard deviation.

The values of the yield strength measured for the different aged samples at other times are shown in Figure 8. A logistic fitting, as developed in our previous study [24], was made on the PLA aged at room temperature, obtaining the following parameters: A = 0.132; B = −0.0477 days−1; $S_{\infty }$ = 64.2 MPa, where A is the ageing potential, B is the ageing rate and $S_{\infty }$ is the property at an infinite time of ageing corresponding to the steady-state, obtaining the following constitutive equation:

(1) $$\sigma_{y}\;\left(\mathrm{MPa}\right)\;=\;\frac{64.2}{1\;+\;0.132e^{-0.0477t}}$$

where $\sigma_{Y}$ is the yield strength in MPa, and t is the ageing time in days. The yield strength results and the Equation (1) obtained from the logistic fitting are represented in Figure 8. For a better visualisation to compare the data between the different ageing methods, a bar diagram is provided in Figure 9.

From the logistic fitting shown in Figure 8, it can be observed that the stabilisation times are expected to be around 100 days, which is coherent with our previous work [24]. Regarding both Figure 8 and Figure 9, it is observed that the yield strength has remained stable for the frozen samples concerning the properties at one day of natural ageing after being printed, as expected from the DSC results in which it is shown that the material is not ageing at −24 °C. However, it has also been observed that the freezing and defrosting process, with or without desiccant, has not damaged the samples, something critical that could not be assessed with the previous results from the DSC.

#### 3.3.2. Elastic Modulus

**Figure 10:**
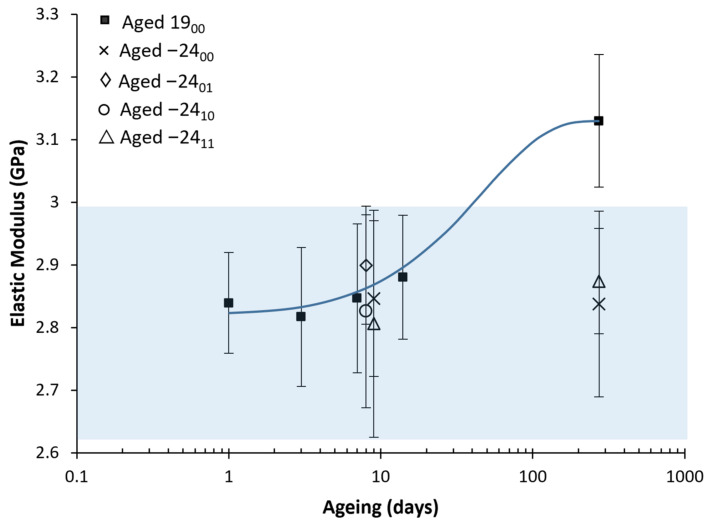
The average value of the elastic modulus with its mean square error vs ageing. The reference material aged at room temperature has bold marks and its logistic fitting (line). For better visualisation, samples aged to nine days are separated into 8 and 9 days in the graph, but all correspond with nine days. The shadow area shows the frozen sample value interval. Six samples were measured for each ageing conditions and error bars are the standard deviation.

**Figure 11:**
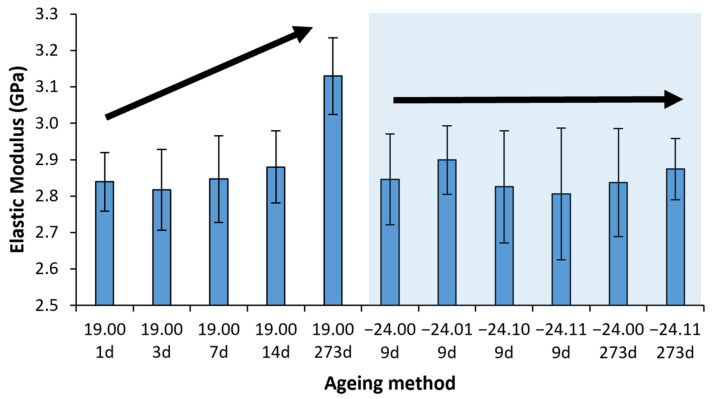
Bar diagram with the average value of the elastic modulus with its mean square error versus the ageing time. The Shadow area shows the frozen sample value interval. Six samples were measured for each ageing conditions and error bars are the standard deviation.

The following results were obtained regarding the Elastic Modulus, shown in Figure 10 and Figure 11. Again, the results were adjusted to the logistic fitting developed in [24], obtaining parameters: A = 0.113; B = −0.023 days−1; $S_{\infty }$ = 3.13 GPa, where A is the ageing potential, B is the ageing rate and $S_{\infty }$ is the property at an infinite time of ageing corresponding to the steady-state, obtaining the following constitutive equation:

(2) $$E\;\left(\mathrm{GPa}\right)\;=\;\frac{3.13}{1\;+\;0.113e^{-0.023t}}$$

where E is the elastic modulus in GPa, and t is the ageing time in days. The experimental results for the elastic modulus and the Equation (2) obtained from the logistic fitting are represented in Figure 10. For better visualisation and comparison of the ageing-defrosting methods, a bar diagram is provided in Figure 11.

The elastic modulus has remained stable through the freezing processes, independently of the presence or not of desiccant. However, it has increased through natural ageing, coherent with our previous work [24]. Although only a slight increase was observed, this is coherent with the also slight increase in the yield strength, which can be made by directly comparing the ageing potential (A) values on Equations (1) and (2) obtained from the logistic fittings.

#### 3.3.3. Elongation at Break and Necking Formation

**Figure 12:**
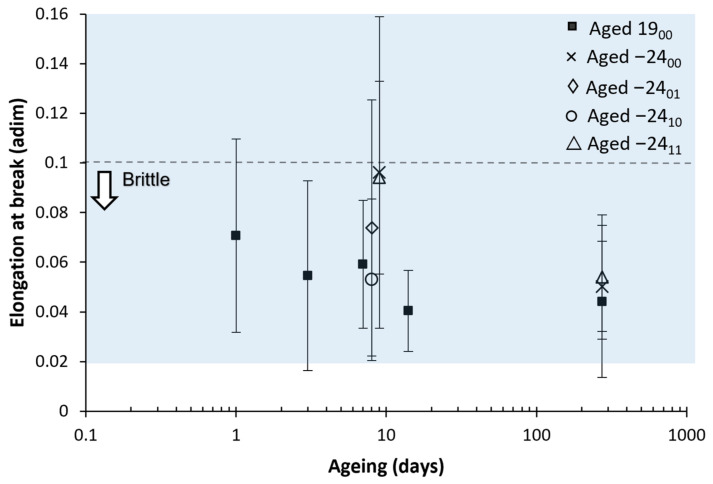
The average value of the elastic modulus with its mean square error versus the ageing. The shadow area shows the frozen sample value interval. For better visualisations, samples aged nine days are separated into 8 and 9 days in the graph, but all correspond with nine days. Six samples were measured for each ageing conditions and error bars are the standard deviation.

**Figure 13:**
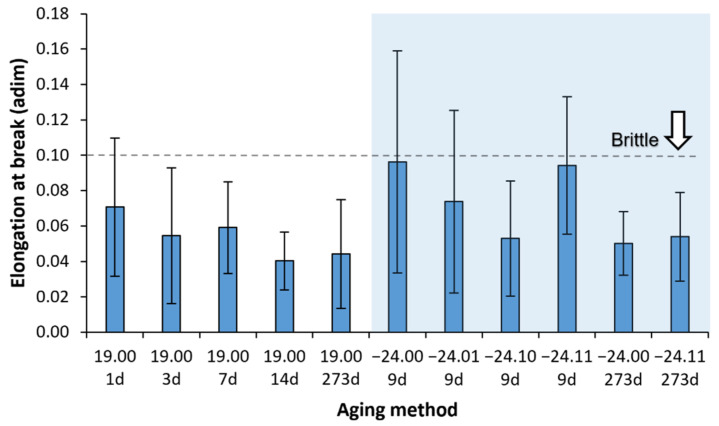
A bar diagram with the average elongation at break with its mean square error versus the ageing time. The shadow area shows the frozen sample value interval. Six samples were measured for each ageing conditions and error bars are the standard deviation.

**Figure 14:**
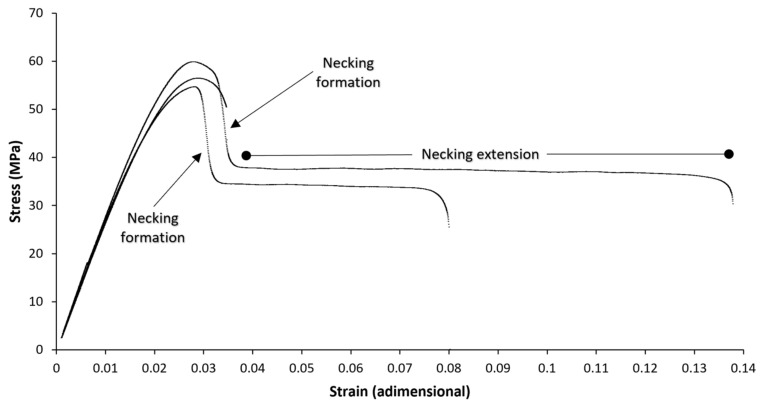
Example of tensile tests with and without necking formation. Notice the long strain for some necking extensions, which has induced considerable standard deviations in the average elongation at break.

The elongation at break results are represented in Figure 12 and Figure 13. The big error bars observed are related to the formation of a necking. When the necking occurs, the elongation at break extended from 3% (0.03) up to 17% (0.17) in this study. Although the necking is useful in the practice for easily detecting a damaged component before it fails, it is a handicap for precisely determining the elongation at break. An example of a tensile test with necking is presented in Figure 14 to visualise the error induced by the necking. PLA is a fragile material, with typical elongation at the break below 10% (0.10), coherent with our results presented. However, the formation or not of a necking was studied to provide further information.

**Figure 15:**
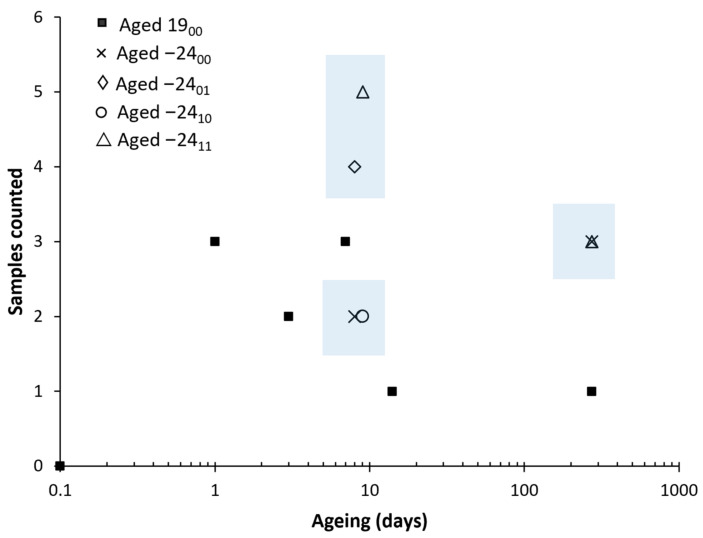
Counts of necking formation. The vertical axis represents how many samples have had a necking out of the six samples tested per material and condition. The shadow area shows the frozen samples. Six samples were measured for each ageing conditions and error bars are the standard deviation.

**Figure 16:**
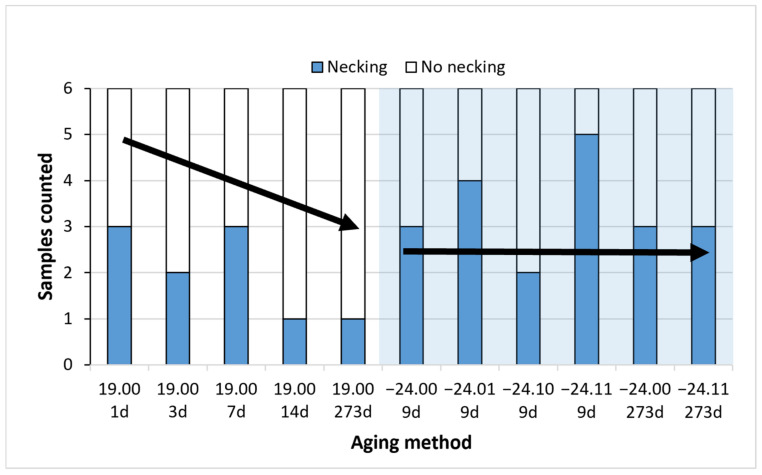
Counts of necking formation. The blue one represents how many samples have had a necking out of the six samples tested per material and condition. Six samples were measured for each ageing conditions and error bars are the standard deviation.

As necking formation has induced this high standard deviation values on the elongation at break, the number of samples that presented necking was counted. This is motivated as necking formation is less likely to happen on aged samples from our experience in our previous study [24]. A total of 6 pieces were tested for each ageing condition. The results are presented in Figure 15 and Figure 16.

It can be observed how natural ageing decreases the formation of the necking. Samples aged at room temperature up to 14 days and 273 days showed only one necking on one of the samples. However, on the frozen samples, almost all the ageing times have shown three or more samples with necking. This clearly indicates that the formation of the necking, related to the microstructure of the PLA and the ease of the polymer chains to move and rearrange during the test, have remained stable with all the freezing–defrosting conditions. This is directly related with the enthalpic relaxation observed in the DSC diagrams.

It is interesting to note how samples aged at 273 days, both aged at room temperature or frozen, demonstrated similar average elongation values at the break. The samples frozen were more prone to form a neck as they had not aged. However, the only neck formed on the specimen at 273 days elongated up to 11%; meanwhile, those necks formed on samples frozen for 273 days elongated between 4% to 9%.

As necking was observed to happen at elongations around 3.0% to 4.0%, it can be observed that a low elongation of the necking has happened on the frozen samples, explaining the similar average value for the stretching. The lower extension of the necking on samples frozen up to 273 days might indicate that there has been some ageing. Still, considering all the thermal and the rest of the mechanical data, the material has remained stable by freezing it. No differences were found regarding the use or not of desiccant.

Although PLA is highly sensitive to relative humidity [31], the humidity levels without desiccant on freezing and defrosting are not crucial for this anti-ageing process. This is an exciting result, as it simplifies the process of stopping the ageing of the PLA by simply freezing the samples at −24 °C inside a zip bag.

Summarising the mechanical properties, they have remained unaltered at −24 °C, and the presence of desiccant has not influenced the results. This result was expected from the thermal study, as mechanical and thermal properties are intimately related [33,34]. However, some mechanical properties are not only associated with the thermal properties but with the geometry and defects of the sample, and it was demonstrated that freezing the PLA has not produced any mechanism that hampers the mechanical properties, such as the formation of surface cracks or the decrease of the M_w_. Again, it can be observed how the PLA aged at room temperature has increased its properties, proving that freezing the PLA has effectively stopped the ageing.

### 3.4. Microstructural and Fractographical Analysis

The samples’ fractographies show more evidence that the anti-ageing method has not damaged the material. This can be observed regarding the similarity among all the fracture surfaces. When the material ages, its properties change and how the material breaks [24]. The fact that there was no change even after 273 days of freezing indicates that the material was kept stable by freezing it.

This PLA had relatively high mechanical properties after being printed. Thus, only a fragile behavior was observed regarding the flatness of the surface crack, which is coherent with our previous results [24]. The most exciting findings here are that the fractographies were similar in all the materials and that freezing the PLA does not affect the PLA’s behavior once defrosted and tested.

**Figure 17:**
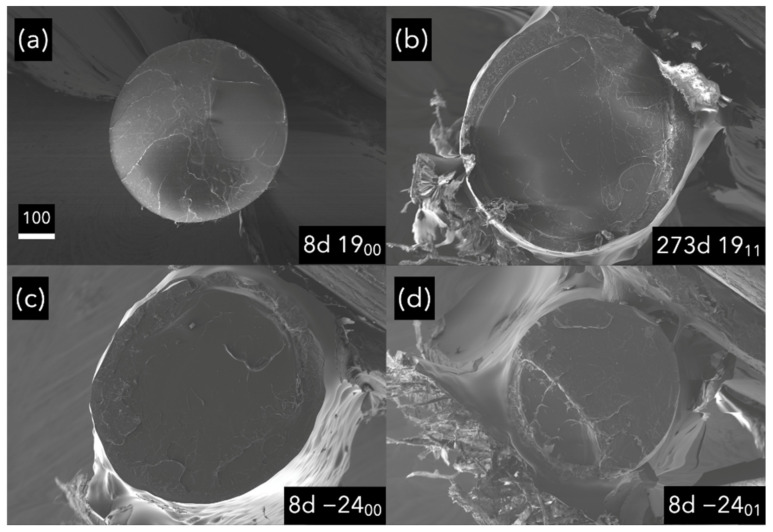

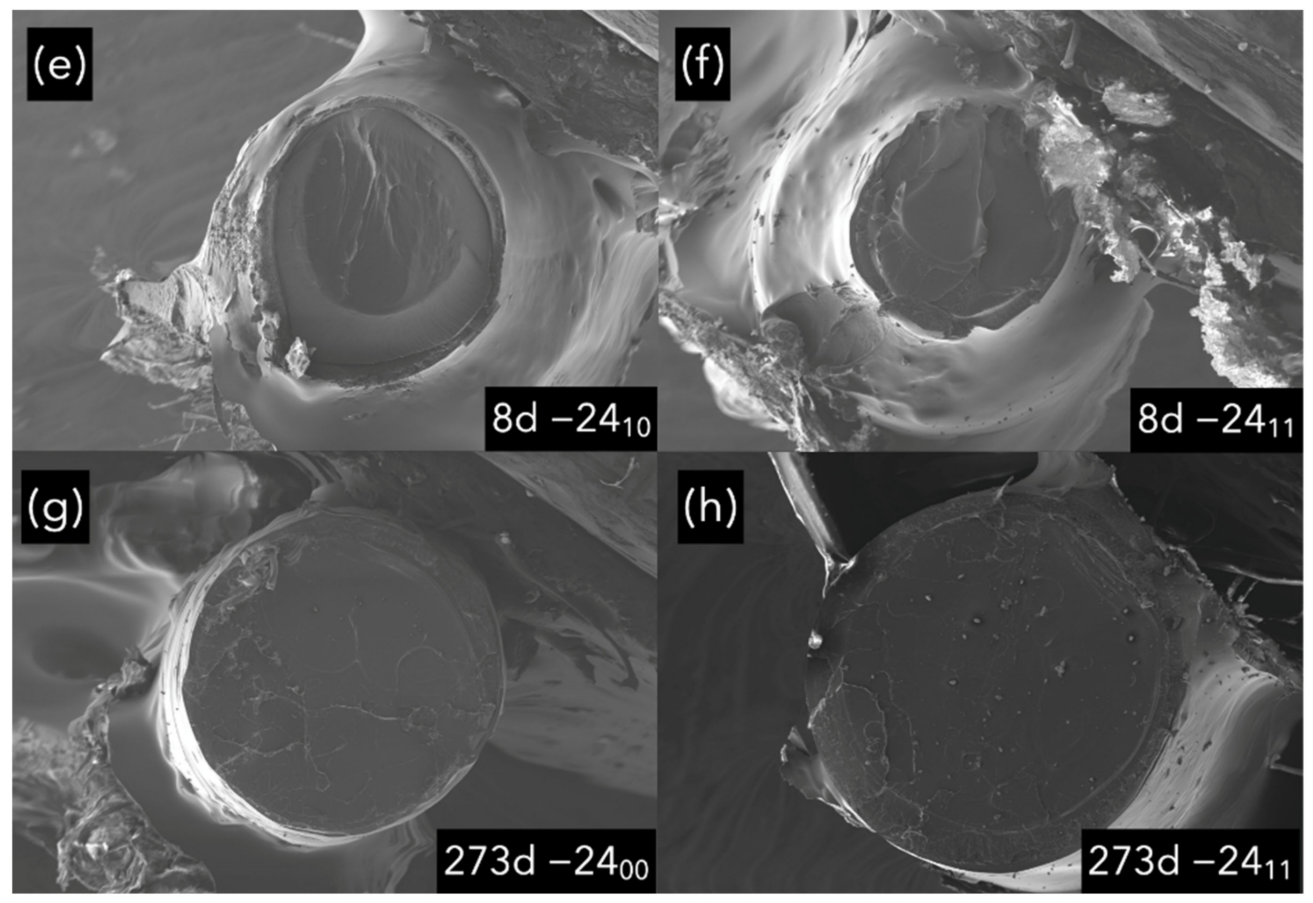
Samples without necking. Scale in microns. (**a**,**b**), samples aged at room temperature; (**c**–**h**), samples aged at −24 °C.

Regarding the fractographical analysis in Figure 17, all the images have shown a similar fracture, indicating that the fracture mechanism was the same for the samples aged at room temperature and frozen. This is important as it provides more evidence that freezing the PLA has not affected the PLA structure, or at least not significantly produced any difference at the micro or the macro scale. It also proves that desiccant is unnecessary for the anti-ageing process inside PET zip-bags, making the anti-ageing process easier from an experimental point of view with the security that the PLA is not going to be degraded or aged in the absence of desiccant inside the PET zip bags.

## 4. Conclusions

According to the results presented, let us state this paper’s central and breakthrough conclusion: it is possible to stop PLA ageing by freezing at −24 °C or lower for up to nine months (probably *at Infinitum*) without damaging the material. Additionally, it can be safely performed without desiccant inside zip bags, simplifying the process so that this anti-ageing procedure is cheap, simple, and effective.

The main features of this new procedure are:

1. Ageing was completely stopped by freezing PLA at −24 °C.
2. No mechanical damage was produced in the frozen PLA.
3. The presence or absence of desiccant during the freezing or defrosting process did not introduce measurable changes in the final state, properties, and behavior of the PLA.
4. It is safe to freeze PLA at −24 °C inside PET zip-bags without a desiccant to stop ageing.

## Figures and Tables

**Table 1:** Methods: the freezing and defrosting routes for the PLA.

Method	Ageing Temperature (°C)	Frozen with Desiccant	Defrosted with Desiccant
19_00_	19 ± 3	- *	- *
−24_00_	−24 ± 1	No	No
−24_01_	−24 ± 1	No	Yes
−24_10_	−24 ± 1	Yes	No
−24_11_	−24 ± 1	Yes	Yes

* Samples stored with a desiccant inside zip-bags and at room temperature.

**Table 2:** Thermal properties of the extruded 1D PLA. T_g_: glass transition; T_ER_: enthalpic relaxation temperature; ΔH_ER_: enthalpy of enthalpic relaxation; T_CC_: cold crystallisation temperature; ΔH_CC_: enthalpy of cold crystallisation; T_m_: melting temperature; ΔH_m_: enthalpy of melting; Xc%: crystallinity content. Following IUPAC’s convention, positive enthalpy changes indicate that the material absorbs the energy, indicative of an endothermal reaction.

Method	Ageing (Days)	T_g_ (°C)	T_ER_ (°C)	ΔH_ER_ (J/g)	T_cc_ (°C)	ΔH_cc_ (J/g)	T_m_ (°C)	ΔH_m_ (J/g)	Xc%
19_00_	1	53.9	56.8	0.0	124	−3.1	150	3.7	<1.5
19_00_	3	55.9	58.8	0.7	125	−2.1	150	2.8	<1.5
19_00_	7	57.2	59.9	1.3	124	−2.7	150	3.7	<1.5
19_00_	240	61.0	63.2	6.1	118	−6.7	149	5.1	<1.5
−24_00_	8	52.1	56.9	0.0	123	−3.6	150	3.6	<1.5
−24_00_	273	51.7	56.1	0.0	119	−4.1	150	4.7	<1.5
−24_01_	8	52.6	57.6	0.0	124	−1.9	150	2.3	<1.5
−24_10_	8	53.7	58.3	0.0	122	−2.8	151	3.3	<1.5
−24_11_	8	53.6	58.2	0.0	125	−2.0	151	2.3	<1.5
−24_11_	273	51.5	56.2	0.0	125	−2.6	151	3.5	<1.5

## Data Availability

Not applicable.
